# Validity of a multi-context sitting questionnaire across demographically diverse population groups: AusDiab3

**DOI:** 10.1186/s12966-015-0309-y

**Published:** 2015-12-04

**Authors:** Bronwyn K Clark, Brigid M Lynch, Elisabeth AH Winkler, Paul A Gardiner, Genevieve N Healy, David W Dunstan, Neville Owen

**Affiliations:** The University of Queensland, School of Public Health, Brisbane, Queensland Australia; Cancer Epidemiology Centre, Cancer Council Victoria, Melbourne, Australia; Melbourne School of Population and Global Health, Faculty of Medicine, Dentistry and Health Sciences, The University of Melbourne, Melbourne, Australia; Baker IDI Heart and Diabetes Institute, Melbourne, Victoria Australia; Mater Research Institute – The University of Queensland, Brisbane, QLD Australia; Curtin University, School of Physiotherapy, Perth, Western Australia Australia; School of Exercise and Nutrition Sciences, Deakin University, Burwood, VIC Australia; Department of Epidemiology and Preventive Medicine, Monash University, Melbourne, VIC Australia; School of Sport Science, Exercise and Health, The University of Western Australia, Perth, WA Australia; Central Clinical School, Monash University, Melbourne, VIC Australia; Mary MacKillop Institute for Health Research, Australian Catholic University, Melbourne, VIC Australia

**Keywords:** activPAL, Demographics, Sedentary, Epidemiology, Measurement

## Abstract

**Background:**

Sitting time questionnaires have largely been validated in small convenience samples. The validity of this multi-context sitting questionnaire against an accurate measure of sitting time is reported in a large demographically diverse sample allowing assessment of validity in varied demographic subgroups.

**Methods:**

A subgroup of participants of the third wave of the Australian Diabetes, Obesity, and Lifestyle (AusDiab3) study wore activPAL3™ monitors (7 days, 24 hours/day protocol) and reported their sitting time for work, travel, television viewing, leisure computer use and “other” purposes, on weekdays and weekend days (*n* = 700, age 36-89 years, 45 % men). Correlations (Pearson’s r; Spearman’s ρ) of the self-report measures (the composite total, contextual measures and items) with monitor-assessed sitting time were assessed in the whole sample and separately in socio-demographic subgroups. Agreement was assessed using Bland-Altman plots.

**Results:**

The composite total had a correlation with monitor-assessed sitting time of r = 0.46 (95 % confidence interval [CI]: 0.40, 0.52); this correlation did not vary significantly between demographic subgroups (all >0.4). The contextual measure most strongly correlated with monitor-assessed sitting time was work (ρ = 0.25, 95 % CI: 0.17, 0.31), followed by television viewing (ρ = 0.16, 95 % CI: 0.09, 0.24). Agreement of the composite total with monitored sitting time was poor, with a positive bias (B = 0.53, SE 0.04, *p* < 0.001) and wide limits of agreement (±4.32 h).

**Conclusions:**

This multi-context questionnaire provides a total sitting time measure that ranks participants well for the purposes of assessing health associations but has limited accuracy relative to activPAL-assessed sitting time. Findings did not differ in demographic subgroups.

**Electronic supplementary material:**

The online version of this article (doi:10.1186/s12966-015-0309-y) contains supplementary material, which is available to authorized users.

## Background

Evidence is accumulating on the risks to health (including premature mortality) posed by prolonged periods of time spent in sedentary behaviours [1, 2], defined as sitting or reclining, and expending less than 1.5 metabolic equivalents (METs) of energy during waking hours [3]. Much of this evidence has been derived from studies examining overall sitting throughout the day or specific sedentary behaviours, such as television (TV) viewing time [4]. In order to understand and influence this adverse exposure, specific domains (e.g. occupational) and/or behaviours (e.g. TV viewing) need to be taken into account [5]. Measurement devices can assess duration and time-of-day when the sedentary time occurs; however, these devices typically do not measure the context [6] and may be expensive in large scale studies. There have been recent developments in methods to identify objectively the relevant contexts in which sedentary behaviors take place through, for example, the use of Global Positioning System devices, wireless location systems and wearable cameras, although these have had difficulty with obtaining usable data particularly inside buildings [7]. Multi-item self-report measures that assess sedentary time spent in particular contexts may provide a cost effective alternative. It is imperative, however, that the properties of such measures are assessed, given the susceptibility of self-report measures to recall error and bias [8].

Many validity studies for sitting time questionnaires have been carried out in small convenience samples, not representative of the general population (e.g. university staff) [6]. Previous studies have shown that accuracy of sitting time recall can vary by age, gender and education [9–12], and, therefore, it is important to assess the validity of questionnaires across different population subgroups for use in public health research.

The third wave of the Australian Diabetes, Obesity, and Lifestyle (AusDiab) study included past-week recall questions asked across a comprehensive array of sedentary domains and behaviours, and collection of objective sitting time data (via activPAL) [13, 14] in a large sub-sample. This presented the opportunity to further understand the measurement properties of the questionnaire. Specifically, we examined the validity of the composite self-reported sitting time relative to sitting time measured by activPAL3™ monitors (monitor-assessed sitting) in terms of agreement and correlations, along with differences between demographic subgroups in relative validity. We also tested the associations with monitor-assessed total sitting time of the various sitting contexts, in all participants, workers and in non-workers.

## Methods

### Data source

The AusDiab baseline study (*n* = 11,247) was initially conducted during 1999 to 2000 to examine the prevalence of diabetes and its risk factors in the Australian population [15]. The third survey (AusDiab3) took place in 2011/12 and 4,614 participants (45 % of those potentially eligible from the baseline sample) attended the onsite testing and answered the questionnaires. Detailed methods of sample recruitment and data collection for the AusDiab study have been described elsewhere [15, 16]. The study was approved by the Ethics Committee of the International Diabetes Institute and The Alfred Health Human Ethics Committee (no. 39/11), and written informed consent was obtained from all participants.

AusDiab3 used the same TV viewing time questions as in prior surveys and added new questions on sitting for work, transport, leisure-time computer use and “other” sitting. AusDiab3 also added an activity monitor assessment in a subsample of eligible participants, who were recruited from on-site attendees at 46 sites across Australia [16]. Each day, participants for the activity monitor sub-study were invited consecutively, beginning with the first potentially eligible participant (i.e., ambulatory, not already known to be pregnant) until either no more devices were available or five participants had been recruited for that day. Participation in this component required informed written consent, additional to that for participation in the main study. In total 1,014 participants in AusDiab3 were approached to participate, and 782 (77 %) agreed.

### Data collection

On the day of recruitment, participants underwent biochemical, anthropometric and behavioural assessments as part of the larger set of AusDiab3 survey procedures. At this visit, the questionnaires were administered by trained interviewers and the activity monitors were either attached by research personnel or self-attached and checked by research staff.

### Measures

#### Referent assessment

Objective data on time spent sitting or lying (collectively referred to here as sitting) were collected using the valid and responsive [14, 17, 18] activPAL3™ activity monitor (PAL Technologies Limited, Glasgow, UK; see Table, Supplemental Digital Content 1 which details methods for the activPAL3™ monitor). The monitor was secured onto the right anterior thigh with a hypoallergenic patch. Participants were asked to wear the monitor continuously (24 h/day) for the seven days following the onsite assessment and to report in a diary all wake up, sleep and monitor removal times (if any). Monitor data were processed in SAS™ 9.3 (SAS Institute Inc., Cary NC; see Additional file 1). Periods spent sleeping or not wearing the monitor, and invalid days, were excluded. Time spent sitting/lying down was totalled for each day and then averaged for all days, weekdays and weekend days deemed valid. Valid days were defined as days with monitor removal <20 % of waking hours, and with ≥10 hours estimated waking wear time (when sleep/wake were not reported in the diary). Sitting time was multiplied by a correction factor (waking hours/worn waking hours) to estimate each individual’s sitting time over the entire waking day (not only the waking wear period).

#### Self-reported sitting time

Participants were asked to report sitting time over the past seven days, separately for weekdays and weekend days, across five contexts (work, transport, TV viewing, leisure time computer use and “other” sitting; see Additional file 2, Sitting time questions from AusDiab3). The periods of recall and activPAL wear did not overlap as the monitors were provided on the same day the questionnaire was administered. Total times over the recall period were converted to average time per day by dividing by five (weekdays), two (weekends), or seven (overall [weekday + weekend]). Such daily averages were calculated for each sitting time item, and the sum of all items (termed composite self-report sitting time).

### Other measures

Socio-demographic data collected in the interviewer-administered questionnaire included: age; gender; education; work status; marital status; annual household income; and, area of residence (categorised as per Table 1). Moderate- to vigorous-intensity physical activity (MVPA) was determined using the Active Australia Survey (AAS), a validated and reliable questionnaire [19, 20], by summing the time spent over the last week in walking, other moderate and vigorous physical activity; (vigorous time multiplied by two as per AAS procedures) [21]. Physical activity status was then categorised as none (0 mins/week), insufficient (>0– < 150 mins/week) and sufficient (≥150 mins/week) according to Australian guidelines [22]. Participants reported whether the week recalled in the physical activity and sedentary behaviour parts of the questionnaire was a “typical week” for them or not. Body Mass Index (BMI) was calculated using measured height and weight (protocol previously described) [16] and was categorised as normal or underweight (<25 kg/m^2^), overweight (≥25 to <30 kg/m^2^) or obese (≥30 kg/m^2^).

**Table 1:** Total sitting time (h/day) assessed by a 10-item, past seven-day recall questionnaire versus by activPAL monitor in Australian adults aged >35 years (*n* = 700)

Characteristic	*n*	Mean (SD) sitting time, h/day		Mean (SD) difference sitting time	r (95 % CI) Self-report vs activPAL	Interaction
		Self-report	activPAL			
Whole Sample	700	6.85 (2.69)	8.86 (1.81)	2.01 (2.45)	0.46 (0.40, 0.52)	
Gender						
Women	388	6.43 (2.47)	8.55 (1.81)	2.12 (2.26)	0.47 (0.39, 0.55)	*p* = 0.69
Men	312	7.36 (2.86)	9.24 (1.74)	1.87 (2.66)	0.42 (0.32, 0.51)	F = 0.16, df = 1
Age group						
35-49 years	125	7.02 (2.80)	8.78 (1.78)	1.76 (2.48)	0.49 (0.35, 0.61)	*p* = 0.89
50-64 years	378	7.09 (2.75)	8.74 (1.86)	1.65 (2.44)	0.50 (0.42, 0.57)	F = 0.11, df = 2
≥65 years	197	6.27 (2.40)	9.13 (1.70)	2.86 (2.24)	0.45 (0.33, 0.55)	
BMI category						
Normal or underweight <25 kg/m^2^)	225	6.54 (2.46)	8.56 (1.69)	2.02 (2.31)	0.43 (0.32, 0.53)	*p* = 0.77
Overweight (≥25– < 30 kg/m^2^)	302	6.89 (2.75)	8.78 (1.80)	1.90 (2.43)	0.49 (0.40, 0.57)	F = 0.27, df = 2
Obese (≥30 kg/m^2^)	173	7.18 (2.87)	9.37 (1.87)	2.19 (2.65)	0.44 (0.31, 0.55)	
Physical activity category						
None (0 min/week)	63	7.23 (2.93)	9.42 (1.72)	2.20 (2.73)	0.43 (0.20, 0.61)	*p* = 0.68
Insufficient (>0 – <150 min/week)	160	7.17 (2.78)	9.07 (1.93)	1.91 (2.56)	0.45 (0.32, 0.67)	F = 0.38, df = 2
Sufficient (≥150 min/week)	477	6.69 (2.61)	8.71 (1.76)	2.02 (2.37)	0.47 (0.39, 0.53)	
Work status						
Full-time work	259	7.75 (2.80)	9.15 (1.88)	1.40 (2.52)	0.48 (0.38, 0.57)	*p* = 0.91
Part-time work	151	6.22 (2.39)	8.42 (1.78)	2.20 (2.33)	0.42 (0.27, 0.54)	F = 0.10, df = 2
Not in paid work	284	6.39 (2.52)	8.82 (1.72)	2.43 (2.34)	0.44 (0.34, 0.53)	
Highest qualification						
Year 12 or less	205	6.25 (2.38)	8.63 (1.85)	2.38 (2.27)	0.45 (0.33, 0.55)	*p* = 0.38
Certificate or diploma	322	6.72 (2.63)	8.83 (1.78)	2.11 (2.45)	0.44 (0.35, 0.52)	F = 0.76, df = 1
Degree or post graduate	169	7.80 (2.89)	9.20 (1.77)	1.39 (2.56)	0.48 (0.36, 0.59)	
Income						
AU$1500+/week	172	7.38 (2.93)	9.23 (1.75)	1.84 (2.55)	0.50 (0.38, 0.61)	*p* = 0.93
AU$800-1499/week	244	7.00 (2.75)	8.78 (1.79)	1.78 (2.40)	0.46 (0.35, 0.55)	F = 0.07, df = 2
<AU$800/week	277	6.37 (2.52)	8.73 (1.84)	2.36 (2.39)	0.44 (0.34, 0.53)	
Marital status						
Married or de facto	539	6.79 (2.64)	8.76 (1.78)	1.96 (2.39)	0.47 (0.40, 0.53)	*p* = 0.56
Not married or de facto	161	7.02 (2.84)	9.18 (1.86)	2.16 (2.63)	0.44 (0.31, 0.56)	F = 0.35, df = 1
Area of residence						
Capital city	438	6.94 (2.73)	8.93 (1.85)	1.99 (2.44)	0.49 (0.41, 0.56)	*p* = 0.27
Not capital city	262	6.69 (2.60)	8.73 (1.74)	2.04 (2.46)	0.42 (0.31, 0.51)	F = 1.21, df = 1

Missing data for work status (*n* = 6), highest qualification (*n* = 4), and income (*n* = 7). Standard deviation (SD), Pearson’s correlation (r), 95 % confidence interval (95 % CI). P for difference between groups in association of self-report with monitor-assessed total sitting time (linear regression)

### Statistical analyses

Analyses were conducted in SPSS version 22.0 (IBM Corporation, Armonk, NY) with statistical significance set at *p* < 0.05 (two-tailed). The multistage sampling led to only a very average design effect (1.3); statistics were reported as per a simple random sample. Only participants who provided at least four valid days of monitor data (including at least one weekend day) (*n* = 711), completed the sitting time questionnaire (*n* = 702) and whose total self-reported sitting time was plausible (≤18 hours/day) (*n* = 700) were included in analyses. Analyses did not exclude the 129 participants (18 %) who reported their recall period was not “typical” for them as relative validity (r = 0.54) was not worse for them than for other participants (r = 0.45). Of the included participants 595 participants [85 %] wore the activPAL for seven valid days.

The associations between self-reported and monitor-assessed sitting time over the whole week, for weekdays, and for weekend days were reported in terms of Pearson’s correlations (r) with 95 % confidence intervals (95 % CI) performed in the whole sample and within various demographic subgroups. Linear regression models with interaction terms (self-report sitting time x demographic characteristic) examined differences between demographic subgroups in associations of self-report sitting with monitor-assessed sitting time. Associations of individual sitting time items with overall monitor-assessed sitting time in the total sample, and specifically in workers and non-workers, were tested using Spearman’s correlations (ρ). Strength of correlations are described according to the criteria established by Cohen: >0.5 large; 0.5–0.3 moderate; 0.3–0.1 small; and, <0.1 insubstantial [23].

Agreement between composite self-report and monitor-assessed sitting time was examined using the method outlined by Bland and Altman [24], with the plot displaying mean difference (MD) and limits of agreement (LoA; +/-1.96 × SD). Linear regression was used to check whether the MD and LoA varied across average values of self-report and monitor-assessed sitting time ([composite self-report sitting + monitor-assessed sitting]/2). Agreement was examined over all days, for weekdays and for weekend days.

## Results

The included sample (*n* = 700) covered participants of ages 36 to 89 years (mean age = 59 years). Most (82 %) reported that the sitting time responses they had provided were indicative of a typical week. Participants were awake (and wearing the monitor) for an average of 15.8 h/day (SD = 1.08) of which 56 % was recorded as sitting or lying down by the activPAL monitor. The included sample was not significantly different to the other AusDiab participants with respect to baseline characteristics of gender, area of residence and work status, but they were younger (*p* < 0.001) and more likely to be married or in a defacto relationship (*p* < 0.01), have a post high school qualification (*p* < 0.001), be born in Australia (*p* < 0.01), be in the normal BMI range (≥18.5- < 25 kg/m^2^, *p* < 0.001), and less likely to have low household income (<AU$800/week, *p* < 0.001) and to report MVPA of 150 minutes or more per week (*p* < 0.01) (see Additional file 3, Characteristics of excluded and included participants at AusDiab1).

### Composite total sitting time

The correlation between self-report sitting time and monitor-assessed sitting time was moderate overall (r = 0.46, 95 % CI: 0.40, 0.52) and consistently moderate (r = 0.42 to 0.50) across various population subgroups (Table 1). A further examination within the oldest group (≥65 years) suggested a weaker correlation of self-report with monitor-assessed sitting within those aged ≥75 years (*n* = 54; r = 0.23, 95 % CI: -0.04, 0.47) than those aged 65 to <75 years (*n* = 143; r = 0.52, 95 % CI: 0.39, 0.63; interaction *p* = 0.046, F = 4.024, df = 1), albeit based on low numbers of participants.

### Context-specific sitting time

Correlations with monitor-assessed sitting time are shown in Table 2, overall and separately for weekdays and weekend days. Correlations between self-report and monitor-assessed total sitting time were moderate for weekday totals (r = 0.49; 95 % CI: 0.43, 0.54) and weak for weekend day totals (r = 0.25; 95 % CI: 0.19, 0.32), even within those with two weekend days of activPAL wear (*n* = 648; r = 0.26; 95 % CI: 0.19, 0.33). Sitting time reported in each context individually had only small or insubstantial correlations with total monitored sitting time, that were typically statistically significant, except for transport sitting, and for “other” sitting. The highest correlations with total monitored sitting overall across the week, on weekdays, and on weekend days respectively were observed for overall work sitting (ρ = 0.25; 95 % CI: 0.17, 0.31), weekday work sitting (ρ = 0.33; 95 % CI: 0.26, 0.40) and weekend TV viewing (ρ = 0.23; 95 % CI 0.16, 0.30). The same was the case within workers (see Additional file 4), while within non-working participants, TV viewing time was the strongest correlate of monitor-assessed sitting time overall across the week, on weekdays and on weekend days.

**Table 2:** Sitting time spent in specific contexts (h/day) as recalled over the past seven days and relative validity against monitored sitting time (activPAL) in Australian adults aged >35 years (*n* = 700)

	Overall		Weekday		Weekend day	
	Median (25^th^, 75^th^ percentile)	Correlation (95 % CI)	Median (25^th^, 75^th^ percentile)	Correlation (95 % CI)	Median (25^th^, 75^th^ percentile)	Correlation (95 % CI)
**Questionnaire, h/day**						
Work	0.71 (0.00, 3.57)	**ρ = 0.25 (0.17, 0.31)**	0.83 (0.00, 4.80)	**ρ = 0.34 (0.27, 0.40)**	0.00 (0.00, 0.00)^a^	ρ = 0.07 (0.00, 0.14)
Transport	0.67 (0.33, 1.07)	ρ = 0.07 (-0.01, 0.14)	0.60 (0.31, 1.00)	**ρ = 0.12 (0.05, 0.20)**	0.50 (0.05, 1.00)	ρ = -0.04 (-0.11, 0.04)
TV	1.71 (0.86, 2.50)	**ρ = 0.16 (0.09, 0.24)**	1.70 (0.80, 2.60)	**ρ = 0.09 (0.01, 0.16)**	2.00 (1.00, 2.50)	**ρ = 0.23 (0.16, 0.30)**
Computer	0.43 (0.07, 1.00)	**ρ = 0.14 (0.06, 0.21)**	0.40 (0.00, 1.00)	**ρ = 0.12 (0.05, 0.19)**	0.25 (0.00, 1.00)	**ρ = 0.10 (0.03, 0.17)**
“Other” sitting	1.43 (0.86, 2.14)	ρ = 0.06 (-0.02, 0.13)	1.15 (0.64, 2.00)	ρ = -0.01 (-0.09, 0.06)	1.96 (1.00, 2.71)	**ρ = 0.11 (0.04, 0.18)**
Total (sum of above)	Mean 6.85 (SD = 2.69)	**r = 0.46 (0.40, 0.52)**	Mean 7.34 (SD = 3.21)	**r = 0.49 (0.43, 0.54)**	Mean 5.62 (SD = 2.66)	**r = 0.25 (0.19, 0.32)**
**activPAL, h/day**						
Monitored sitting time	Mean 8.86 (SD = 1.81)	-	Mean 8.96 (SD = 1.98)	-	Mean 8.48 (SD = 2.10)	-

Data for sitting times are median (25, 75 %) except where indicated as mean (SD). Spearman’s correlation (ρ), Pearson’s correlation (r), findings in bold are significant. ^a^Only 124 participants reported sitting for work on the weekend with median time of 1.50 h/day (25 %: 0.75, 75 %: 2.50) within those who worked

### Agreement

**Fig. 1:**
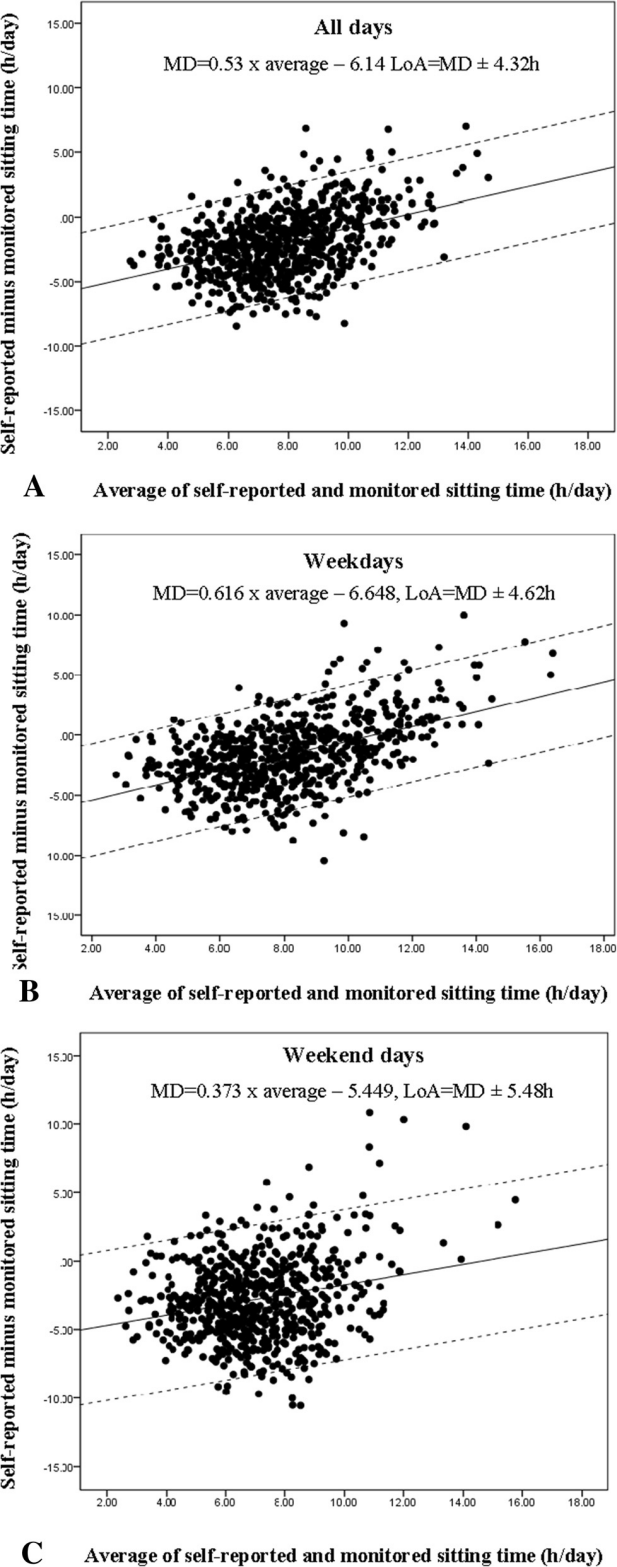
Bland-Altman Plots for total sitting time (h/day) reported over the last seven days across five contexts versus monitored by activPAL (*n* = 700 Australian adults >35 years): overall (**a**); on weekdays (**b**); and, on weekend days (**c**). The solid line represents the mean difference (MD) between the two measures and the dashed lines are the 95 % limits of agreement (LoA) in h/day

The Bland-Altman plot for the agreement in total (composite) sitting time (h/day) assessed by self-report versus monitor is shown in Fig. 1. The difference between the instruments increased significantly with the average of the two measures, but the 95 % limits of agreement were constant, and consistently large, at ± 4.32 h/day (Fig. 1). For all but the very highest levels of average sitting time (>approximately 14 h /day), the questionnaire underestimated relative to the monitor. The difference between the two measures was -2.01 h/day on average (i.e., at 7.85 h/day, mean of self-report and activPAL sitting time). A positive bias, and wide limits of agreement were also seen for weekday and weekend day sitting (Fig. 1).

## Discussion

In this sample of Australian adults, we found that composite self-reported sitting time recalled over the past seven days, measured using a 10-item multi-context questionnaire, was moderately correlated (r = 0.46, 95 % CI: 0.40, 0.52) with monitor-assessed sitting time overall and across a wide range of demographic groups. The self-report measure was an acceptable method to rank participants’ sitting time. This measure is not appropriate when accurate estimates of actual sitting time are required as agreement with monitor-assessed sitting time was poor, both at a group level (mean differences averaging 2 h/day that varied proportionally to average sitting time), and for individuals (>4 h/day limits of agreement).

Mostly, sedentary behaviour questionnaires show small to moderate correlations (predominantly <0.40) with referent measures, typically derived from waist-worn accelerometers (sedentary time <100 counts per minute, vertical axis) that do not measure sitting [6]. Our findings show correlations of r = 0.4 to r = 0.5, despite the recall and device wear weeks being completely separate, which tends to weaken correlations. Weekend day measures may be the most affected, with the least days of monitoring available to minimise the between-day variation. The relative validity in this current study was similar to that observed against activPAL sitting time within overweight breast cancer survivors for total sitting time assessed using the seven-context Past-day Adults Sedentary Time questionnaire (PAST; on recalled day r = 0.58, 95 % CI 0.40, 0.72; for overall weekly sitting time r = 0.36; 95 % CI 0.11, 0.57) [25] and the Dutch version of the SIT-Q_7d, a five-context past week recall questionnaire (ρ = 0.52, *p* < 0.001) with similar contexts to the AusDiab questionnaire but with categorical responses [26].

Previous studies have examined and found differences in the validity of sitting time questionnaires by age, gender, and education [9, 11, 12, 27]. We observed no significant or large differences in relative validity between socio-demographic groups aside from poorer relative validity within older adults (≥75 years) than their younger counterparts. Age-related differences in validity have been observed elsewhere [11]. While this could reflect an age bias in the referent measure, some caution should be exercised when using the questionnaire in adults aged ≥75 years.

Sitting for work and for TV viewing were consistently the two contextual measures most strongly correlated with monitor-assessed total sitting time. Their relative importance seemed to depend on the time period of interest (work was important overall and for weekday sitting while TV viewing was especially important for weekend-day sitting) and participant work status. In non-workers, TV viewing was always the most important context, which was somewhat consistent with a finding from NHANES [28], that the correlation of TV viewing time with total accelerometer-derived sedentary time (<100 cpm) appeared stronger within non-workers than workers.

Agreement between composite self-report and monitor-assessed sitting time was poor, with a positive linear relationship between the difference and average of the two measures. At mean levels of average sitting time, participants reported over two hours less sitting per day than was recorded by the activPAL. This bias is similar to previous findings for sedentary behaviour questionnaires compared to activPAL sitting time [25, 26] and to accelerometer-derived sedentary time (100 cpm) [11] for both single-item sitting time questions [10, 29] and composite measures of sitting time [11, 30]. Limits of agreement were also wide, indicating poor individual accuracy.

Both self-report and monitor-based measures have their own unique place in furthering the science of sedentary behaviour [31]. Self-report measures, such as the AusDiab3 multi-context questionnaire, can provide important behavioural context information, are a cost-effective method of ranking participants, and are useful in assessing relationships between sitting time and health outcomes. However, when accurate sitting time duration is required, monitors are more appropriate in order to provide the necessary precision at both the group and the individual level [13].

A key strength of this study was the comparison self-report questions against an accurate measure of sitting time (activPAL3) [13, 14] in a large and diverse sample of adults. Although not population representative, the sample was much closer to representative than can be said of most validity studies, covering a wide spectrum of Australian adults across multiple geographic locations, and chiefly lacking only adults aged ≤35 years. Findings cannot be generalised to young adults. The validity of individual sitting contexts, such as computer use, may differ for young adults from what we observed. Arguably, this study may have overestimated the validity of the questionnaire as the general reporting abilities may be greater for this group (now involved in the third wave of a longitudinal study) than for the population at large. However, this specific questionnaire had never been administered to the participants in previous waves (with the exception of the TV viewing question). A further limitation of the study design was that sedentary behavior may vary over time, but only a single assessment of each measure was taken, close together in time, but not at the same time. As such our findings likely underestimate what the agreement and correlation would be if our objective and self-report measures were obtained for precisely the same week. Ideally, for behaviors that are likely to vary over time, validity should be assessed for long-term averages [32]. Methods to address this have been developed, involving performing assessments repeatedly, spread across an extended period of time [32]. Additionally, the reliability of this measure was not assessed, although both self-report and device-based measures of sedentary behavior typically show good reliability [6, 33].

## Conclusions

The composite total sitting time derived from these domain- and behaviour-specific sitting questions showed acceptable ability to rank participants relative to monitored sitting time, and this quality extended across different socio-demographic groups. Self-reported total sitting time, however, was not accurate at either the group or individual level. This measure, therefore, may be most suited to assessing associations of sitting time with health outcomes, but would not be useful to accurately estimate total sitting time.

## Additional files

Additional file 1:
**AF 1 IJBNPA AusDiab3 sitting questionnaire validity.**pdf; Details of activity monitor (activPAL) data collection and processing. (PDF 67 kb) Additional file 2:
**AF 2 IJBNPA AusDiab3 sitting questionnaire validity.**pdf; AusDiab3 sitting time questions. (PDF 49 kb) Additional file 3:
**AF 3 IJBNPA AusDiab3 sitting questionnaire validity**.pdf; Comparison of baseline (AusDiab1) characteristics between AusDiab1 participants who ultimately were included (*n* = 700) or not included (*n* = 10547) in the present study, due to sub-study non-selection/non-participation/lack of data, or loss to follow-up (AusDiab1–AusDiab3). (PDF 58 kb) Additional file 4:
**AF 4 IJBNPA AusDiab3 sitting questionnaire validity.**pdf; Sitting time in various contexts recalled over the past seven days and relative validity against total sitting time assessed by activPAL™ within participant in paid work (*n* = 410) and not in paid work (*n* = 284). (PDF 47 kb)
