# Supplementary material for: Validity of a multi-context sitting questionnaire across demographically diverse population groups: AusDiab3
Source: Int J Behav Nutr Phys Act. 2015 Dec 4;12:148. doi: 10.1186/s12966-015-0309-y (PMC4670496; doi:10.1186/s12966-015-0309-y)
Supplement: Additional file 1: — AF 1 IJBNPA AusDiab3 sitting questionnaire validity.pdf; Details of activity monitor (activPAL) data collection and processing. (PDF 67 kb) [file 12966_2015_309_MOESM1_ESM.pdf]

Additional file 1

Table: Details of activity monitor (activPAL) data collection and processing

**Item**	**Details**
Monitor version	activPAL3
Reliability (inter- and intra-instrument) for the device selected	Interdevice reliability for the activPAL device ranged from 0.79 to 0.99 (20).
Validity information for the activity estimates of interest	A mean percentage difference of 0.19% (limits of agreement −0.68% to 1.06%) and 1.4% (limits of agreement -6.2% to 9.1%) between the activPAL monitor and observation for total time spent sitting and standing has been reported (20). Not only do the totals work out correctly, it has been shown that sitting/lying, standing, and walking are correctly identified by the activpal (100% of the time) when they are occurring, with almost perfect agreement between the activPAL and direct observation (kappa=0.98)(3). The activPAL accurately identifies number of steps and walking speed accurately across both low and high speeds (absolute percentage error <1.11%)(30).
Method and location of monitor attachment	Device was waterproofed by covering in nitrile fingercot and wrapped fully in one piece of waterproof dressing (Hypafix transparent). Adhered to mid-thigh anterior aspect using one piece of Hypafix dressing following visual demonstration. Either attached by research staff or self-adhered with attachment checked by research staff. Additional dressings and alcohol wipes supplied for reattachment during wear period.
Wear period and number of days	24 hours/day for 7 consecutive days
activPAL software version	Version 6.4.1
Settings used:<br>Sampling Frequency<br>Minimum sitting period<br>Minimum upright period	All defaults. Selected information below:<br>10Hz (default)<br>10 seconds (default)<br>10 seconds (default)
Diary data collected and details collected	Time woke up, time got up, and any removal times each day
Type of file used for data processing	Events file
Method(s) for estimating wearing time/removing time in bed/sleep	Whole bouts of activity were classed as awake/not and removed/not. Bouts containing mostly (≥50%) minutes reported in the diary to be awake, or removed were initially classed as such. The beginning and ending of sleep periods (and pre-study and post-study removals) were then modified to coincide with

	the first bout / last bout of sitting/reclining or standing ≥20 minutes duration within the initially identified sleeping period. The whole bout classification firstly adjusts the estimation from participants' imprecise times (whole minutes, often rounded off) and secondly provides more plausible estimates as removing the monitor, arising from bed, going to bed etcetera usually entail a change in posture or movement. Unreported sleep /wake times were estimated from the activPAL events files by research staff.
What quality control checks were implemented	All included and excluded data were checked visually (via heatmaps) to identify any instances where it seemed movement during wake was more consistent with sleep or removal and vice versa, or if the monitor appeared to have been worn upside down. Problems were rectified individually based on best consistency between the monitor data and the diary (if available).
Specify type of action taken when data were determined to be invalid	Data deemed invalid (removal and sleep periods, all time on invalid days, selected days that failed the quality control checks) were excluded from analysis of worn waking hours.
Compliance criteria to define a valid day of observation*	Worn for ≥80% of waking hours (when waking hours were reported in the diary)<br>OR<br>Worn for ≥80% of waking hours and ≥10 worn waking hours (when waking hours were estimated from the monitor).
Number and type of days required to be included in final analytic sample	Four or more valid days of data including a weekend day.
Data processing package used and methods used to generate key summary variables	activPAL software version 6.4.1 to create events files.<br>A SAS 9.3 program created by the manuscript authors (EW) was used to perform quality checks and determine valid data
References	1. Berendsen BA, Hendriks MR, Meijer K, Plasqui G, Schaper NC, and Savelberg HH, Which activity monitor to use? Validity, reproducibility and user friendliness of three activity monitors. *BMC Public Health*, 2014; 14(1): p. 749.<br>2. Grant P, Ryan C, Tigbe W, and Granat M, The validation of a novel activity monitor in the measurement of posture and motion during everyday activities. *Br J Sports Med*, 2006; 40(12): p. 992 - 997.<br>3. Ryan CG, Grant PM, Tigbe WW, and Granat MH, The validity and reliability of a novel activity monitor as a measure of walking. *British Journal of Sports Medicine*, 2006; 40(9): p. 779-784.
