# Supplementary material for: Validity of a multi-context sitting questionnaire across demographically diverse population groups: AusDiab3
Source: Int J Behav Nutr Phys Act. 2015 Dec 4;12:148. doi: 10.1186/s12966-015-0309-y (PMC4670496; doi:10.1186/s12966-015-0309-y)
Supplement: Additional file 2: — AF 2 IJBNPA AusDiab3 sitting questionnaire validity.pdf; AusDiab3 sitting time questions. (PDF 49 kb) [file 12966_2015_309_MOESM2_ESM.pdf]

Additional File 2: AusDiab3 sitting time questions

AusDiab: The Australian Diabetes, Obesity and Lifestyle Study

Sitting time

Next I will ask you about particular activities you did last week while sitting down. Again, I want you to think about last ** to yesterday. These questions will ask about the total time you spent sitting for these purposes in the last week.

Sitting for work in the last week

Please estimate the total time during the last week that you spent sitting down as part of your job while at work or working from home, including meal and snack breaks and sitting to do work such as at desk or in meetings, sitting to use the computer at work, and sitting for travel as part of work such as being a taxi driver?

Monday to Friday **(In hours and/or minutes - fill in all circles on answer sheet)**

		hours			minutes

Saturday and Sunday **(In hours and/or minutes - fill in all circles on answer sheet)**

		hours			minutes

Sitting for transport in the last week

Please estimate the total time during the last week that you spent time sitting to travel to or from places? Please include sitting and waiting for transport. Do not include any time you were standing up while travelling or waiting. If you spent sitting for travelling as part of your work, such as being a taxi driver, this should have been included in the question about sitting for work so do not report it here.

Monday to Friday **(In hours and/or minutes - fill in all circles on answer sheet)**

		hours			minutes

Saturday and Sunday **(In hours and/or minutes - fill in all circles on answer sheet)**

		hours			minutes

TV viewing in the last week

Please estimate the total time during the last week that you spent watching TV or videos/DVDs. This is when it was the main activity that you were doing; for example you would not include time when the television was switched on and you were preparing a meal.

Monday to Friday **(In hours and/or minutes - fill in all circles on answer sheet)**

		hours			minutes

Saturday and Sunday **(In hours and/or minutes - fill in all circles on answer sheet)**

		hours			minutes

Computer, internet, electronic games in the last week

Please estimate the total time during the last week that you spent sitting and using the computer. For example, include time spent playing any Playstation, Nintendo, X-Box, computer or handheld console games, and time spent on internet activities. Do not include time spent doing paid work on the computer as this should have been included in the previous questions about sitting for work.

Monday to Friday **(In hours and/or minutes - fill in all circles on answer sheet)**

		hours			minutes

Saturday and Sunday **(In hours and/or minutes - fill in all circles on answer sheet)**

		hours			minutes

Sitting for other purposes in the last week

We are interested in any other sitting or lying down that you may have done during your waking hours in the last week that you have not already told us about. For example this could include sitting for reading or hobbies, socialising with friends or family including time on the telephone eating meals; or listening to music.

Please estimate the total time during the last week that you spent sitting down **NOT** including sitting for work, transport, television viewing, or computer use that you have mentioned in the previous answers?

Monday to Friday **(In hours and/or minutes - fill in all circles on answer sheet)**

		hours			minutes

Saturday and Sunday **(In hours and/or minutes - fill in all circles on answer sheet)**

		hours			minutes
