# Supplementary material for: Validity of a multi-context sitting questionnaire across demographically diverse population groups: AusDiab3
Source: Int J Behav Nutr Phys Act. 2015 Dec 4;12:148. doi: 10.1186/s12966-015-0309-y (PMC4670496; doi:10.1186/s12966-015-0309-y)
Supplement: Additional file 3: — AF 3 IJBNPA AusDiab3 sitting questionnaire validity.pdf; Comparison of baseline (AusDiab1) characteristics between AusDiab1 participants who ultimately were included (n = 700) or not included (n = 10547) in the present study, due to sub-study non-selection/non-participation/lack of data, or loss to follow-up (AusDiab1–AusDiab3). (PDF 58 kb) [file 12966_2015_309_MOESM3_ESM.pdf]

Additional File 3

Table: Comparison of baseline (AusDiab1) characteristics between AusDiab1 participants who ultimately were included (n=700) or not included (n=10547) in the present study, due to sub-study non-selection/non-participation/lack of data, or loss to follow-up (AusDiab1–AusDiab3).

Baseline (AusDiab1) characteristic	Excluded participants	Included participants	p for difference
Participant numbers	10547	700	
Age in years, mean (SD)	51.85 (14.65)	46.71 (10.28)	$p<0.001$
Male	4736 (44.9%)	312 (44.6%)	$p=0.88$
Lives in capital city	6473 (61.4%)	438 (62.6%)	$p=0.55$
Married or defacto relationship	8001 (75.9%)	568 (81.1%)	$p<0.01$
Post-high school qualification	6092 (57.8%)	491 (70.1%)	$p<0.001$
Working full- or part-time	6000 (56.9%)	543 (77.6%)	$p=0.47$
Income <AU$800/week	5869 (55.6%)	277 (39.6%)	$p<0.001$
Country of birth Australia	7975 (75.6%)	571 (81.6%)	$p<0.01$
BMI category of normal (≥18.5–<25 kg/m^2)	3865 (36.7%)	294 (42.0%)	$p<0.001$
Reports ≥150 mins/week LTPA	5386 (51.1%)	410 (48.6%)	$p<0.01$

Data are n (%) except where indicated. Missing data on baseline characteristics for the excluded participants for marital status (n=6), qualifications (n=21), income (n=92), country of birth (n=2) and BMI (n=174); and included participants for income (n=2) and BMI (n=6). Data are not weighted.
