# Supplementary material for: Validity of a multi-context sitting questionnaire across demographically diverse population groups: AusDiab3
Source: Int J Behav Nutr Phys Act. 2015 Dec 4;12:148. doi: 10.1186/s12966-015-0309-y (PMC4670496; doi:10.1186/s12966-015-0309-y)
Supplement: Additional file 4: — AF 4 IJBNPA AusDiab3 sitting questionnaire validity.pdf; Sitting time in various contexts recalled over the past seven days and relative validity against total sitting time assessed by activPAL™ within participant in paid work (n = 410) and not in paid work (n = 284). (PDF 47 kb) [file 12966_2015_309_MOESM4_ESM.pdf]

Additional File 4

Table: Sitting time in various contexts recalled over the past seven days and relative validity against total sitting time assessed by activPAL^{TM} within participant in paid work (n=410) and not in paid work (n=284)

Context	Overall (weekend and weekdays)		Weekdays		Weekend Days	
	**Median (25th, 75th percentile)**	**ρ (95% CI)**	**Median (25th, 75th percentile)**	**ρ (95% CI)**	**Median (25th, 75th percentile)**	**ρ (95% CI)**
**Workers (n=410)**						
Work sitting, h/day	2.29 (0.57, 4.29)	0.43 (0.35, 0.50)	3.00 (0.60, 6.00)	0.51 (0.42, 0.57)	0.00 (0.00, 0.00)	0.11 (0.01, 0.20)
Transport sitting, h/day	0.71 (0.38, 1.14)	0.07 (-0.03, 0.16)	0.70 (0.33, 1.18)	0.11 (0.01, 0.21)	0.50 (0.17, 1.00)	-0.03 (-0.12, 0.07)
TV sitting, h/day	1.43 (0.71, 2.14)	0.09 (-0.01, 0.18)	1.20 (0.60, 2.00)	0.02 (-0.08, 0.12)	1.50 (0.75, 2.50)	0.18 (0.08, 0.27)
Computer sitting, h/day	0.36 (0.07, 0.86)	0.12 (0.02, 0.22)	0.40 (0.05, 1.00)	0.10 (-0.00, 0.19)	0.25 (0.00, 1.00)	0.13 (0.04, 0.23)
"Other" sitting, h/day	1.29 (0.79, 2.00)	0.04 (-0.06, 0.14)	1.00 (0.06, 2.00)	-0.04 (-0.13, 0.06)	1.50 (1.00, 2.50)	0.09 (-0.01, 0.18)
**Not in paid work (n=284)**						
Transport sitting, h/day	0.57 (0.29, 1.00)	0.07 (-0.05, 0.19)	0.60 (0.30, 1.00)	0.13 (0.02, 0.24)	0.50 (0.00, 1.00)	-0.03 (-0.14, 0.09)
TV sitting, h/day	2.00 (1.00, 3.00)	0.31 (0.20, 0.41)*	2.00 (1.00, 3.00)	0.26 (0.15, 0.37)*	2.00 (1.00, 3.00)	0.26 (0.15, 0.37)
Computer, h/day	0.43 (0.00, 1.00)	0.15 (0.03, 0.26)	0.40 (0.00, 1.00)	0.16 (0.05, 0.27)	0.25 (0.00, 1.00)	0.05 (-0.06, 0.17)
"Other" sitting, h/day	1.29 (0.79, 2.00)	0.10 (-0.02, 0.21)	1.60 (1.00, 2.80)	0.07 (-0.05, 0.18)	1.50 (1.00, 2.50)	0.11 (-0.01, 0.22)

* significantly different to association in workers (p for interaction <0.05).
